# The neutrophil-to-lymphocyte ratio, lymphocyte-to-monocyte ratio, and neutrophil-to-high-density-lipoprotein ratio are correlated with the severity of Parkinson’s disease

**DOI:** 10.3389/fneur.2024.1322228

**Published:** 2024-01-23

**Authors:** Fangyi Li, Guomei Weng, Hang Zhou, Wenjie Zhang, Bin Deng, Yuqi Luo, Xi Tao, Mingzhu Deng, Haiqiang Guo, Shuzhen Zhu, Qing Wang

**Affiliations:** ^1^Department of Neurology, Zhujiang Hospital of Southern Medical University, Guangzhou, China; ^2^Department of Neurology, The Affiliated Changsha Central Hospital, Hengyang Medical School, University of South China, Changsha, China; ^3^Department of Neurology, The First People’s Hospital of Zhaoqing, Zhaoqing, China; ^4^Department of Neurological Rehabilitation, Hunan Provincial People’s Hospital, The First Affiliated Hospital of Hunan Normal University, Changsha, China; ^5^Department of Neurology, Brain Hospital of Hunan Province, The Second People’s Hospital of Hunan Province, Changsha, China; ^6^Department of Neurology, Dafeng Hospital of Chaoyang District in Shantou City, Shantou, China

**Keywords:** Parkinson’s disease, neutrophil-to-lymphocyte ratio, lymphocyte-to-monocyte ratio, neutrophil-to-high-density lipoprotein ratio, inflammation

## Abstract

**Background:**

Inflammation plays a pivotal role in the pathogenesis of Parkinson’s disease (PD). However, the correlation between peripheral inflammatory markers and the severity of PD remains unclear.

**Methods:**

The following items in plasma were collected for assessment among patients with PD (*n* = 303) and healthy controls (HCs; *n* = 303) were assessed for the neutrophil-to-lymphocyte ratio (NLR), lymphocyte-to-monocyte ratio (LMR) and neutrophil-to-high-density-lipoprotein ratio (NHR) in plasma, and neuropsychological assessments were performed for all patients with PD. Spearman rank or Pearson correlation was used to evaluate the correlation between the NLR, the LMR and the NHR and the severity of PD. Receiver operating characteristic (ROC) curves were used to evaluate the diagnostic performance of the NLR, LMR and NHR for PD.

**Results:**

The plasma NLR and NHR were substantially higher in patients with PD than in HCs, while the plasma LMR was substantially lower. The plasma NLR was positively correlated with Hoehn and Yahr staging scale (H&Y), Unified Parkinson’s Disease Rating Scale (UPDRS), UPDRS-I, UPDRS-II, and UPDRS-III scores. Conversely, it exhibited a negative relationship with Mini-Mental State Examination (MMSE) and Montreal Cognitive Assessment (MoCA) scores. Furthermore, the plasma NHR was positively correlated with H&Y, UPDRS, UPDRS-I, UPDRS-II and UPDRS-III scores. Moreover, negative associations were established between the plasma LMR and H&Y, UPDRS, UPDRS-I, UPDRS-II, and UPDRS-III scores. Finally, based on the ROC curve analysis, the NLR, LMR and NHR exhibited respectable PD discriminating power.

**Conclusion:**

Our research indicates that a higher NLR and NHR and a lower LMR may be relevant for assessing the severity of PD and appear to be promising disease-state biomarker candidates.

## Introduction

Parkinson’s disease (PD) is a prevalent degenerative neurological condition of the central nervous system that primarily affects middle-aged and elderly individuals (1–3). Its main clinical manifestations include motor retardation, resting tremor, postural instability, and muscle rigidity (4–6). Recent research has indicated that peripheral inflammation plays an important role in the development of PD (7–10). Currently, certain biomarkers can be used to evaluate the severity and progression of PD. These factors include α-synuclein, total tau, and cerebrospinal fluid Aβ40 levels (11, 12). Additionally, serum neurofilament light chain (NFL), glial fibrillary acid protein (GFAP), small extracellular vesicle (sEV), and serum long noncoding RNA (lncRNA) GAS5 levels and TNF-α levels are emerging as promising candidates for disease state biomarkers (13–16). Magnetic resonance imaging (MRI) and positron emission tomography (PET) may help differentiate PD from other atypical parkinsonisms (17). However, the high cost or invasive sampling methods of these techniques limit their wide-scale clinical applicability.

In recent years, the neutrophil-lymphocyte ratio (NLR), lymphocyte-monocyte ratio (LMR) and neutrophil-to-high-density lipoprotein ratio (NHR), platelet-to-lymphocyte ratio (PLR) have been considered useful inflammatory markers for neurological diseases (18, 19). Neutrophils can penetrate epithelial and vessel-wall cell layers and promote the inflammatory response of the body by regulating chemokines (20). T-cell infiltration has been detected in the hippocampus, neocortex, striatal perivascular area, and parenchyma of mice with PD, and lower lymphocyte counts are related to a greater risk of PD (21). Additionally, there are also research findings that with a higher lymphocyte count to be less likely to have prevalent PD (22). The NLR is a rapid and effective indicator of peripheral inflammation, and several studies have shown higher NLR in patients with PD (23). However, the relationship between the NLR and PD incidence remains controversial. A previous study reported a positive correlation between the NLR and disease duration and between the Hoehn and Yahr staging scale (H&Y) score and PD incidence (24), while another study reported conflicting results (23).

The integrity of the blood-brain barrier (BBB) is closely related to the plasma level of high-density lipoprotein cholesterol (25, 26), and plasma high-density lipoprotein cholesterol levels are significantly lower (27) or remain unchanged (28) in patients with PD. It has been shown that NHR levels are significantly greater in patients with PD than in healthy controls (HCs) and that NHR levels are negatively correlated with disease duration (29). Recent studies have shown that patients with PD have dysregulated blood monocytes and exhibit the proinflammatory effects of α-synuclein on monocytes (30). Moreover, the LMR and C-reactive protein (CRP) level may serve as useful predictors of patient prognosis in patients with PD combined with rapid eye movement sleep behavior disorder (31). However, the relationship between the LMR and the severity of PD has not been determined.

Given that the inflammatory response actively participates in the onset of PD, identifying a blood-based inflammatory indicator is necessary for assessing and monitoring the progression of PD (32). However, research on the associations between the severity of PD and these indicators, including the NLR, the LMR and the NHR, is insufficient, and further investigations are needed. Therefore, our study was designed to explore the potential of these factors as indicators for determining the progression of Parkinson’s disease.

## Materials and methods

### Subjects

**Figure 1:**
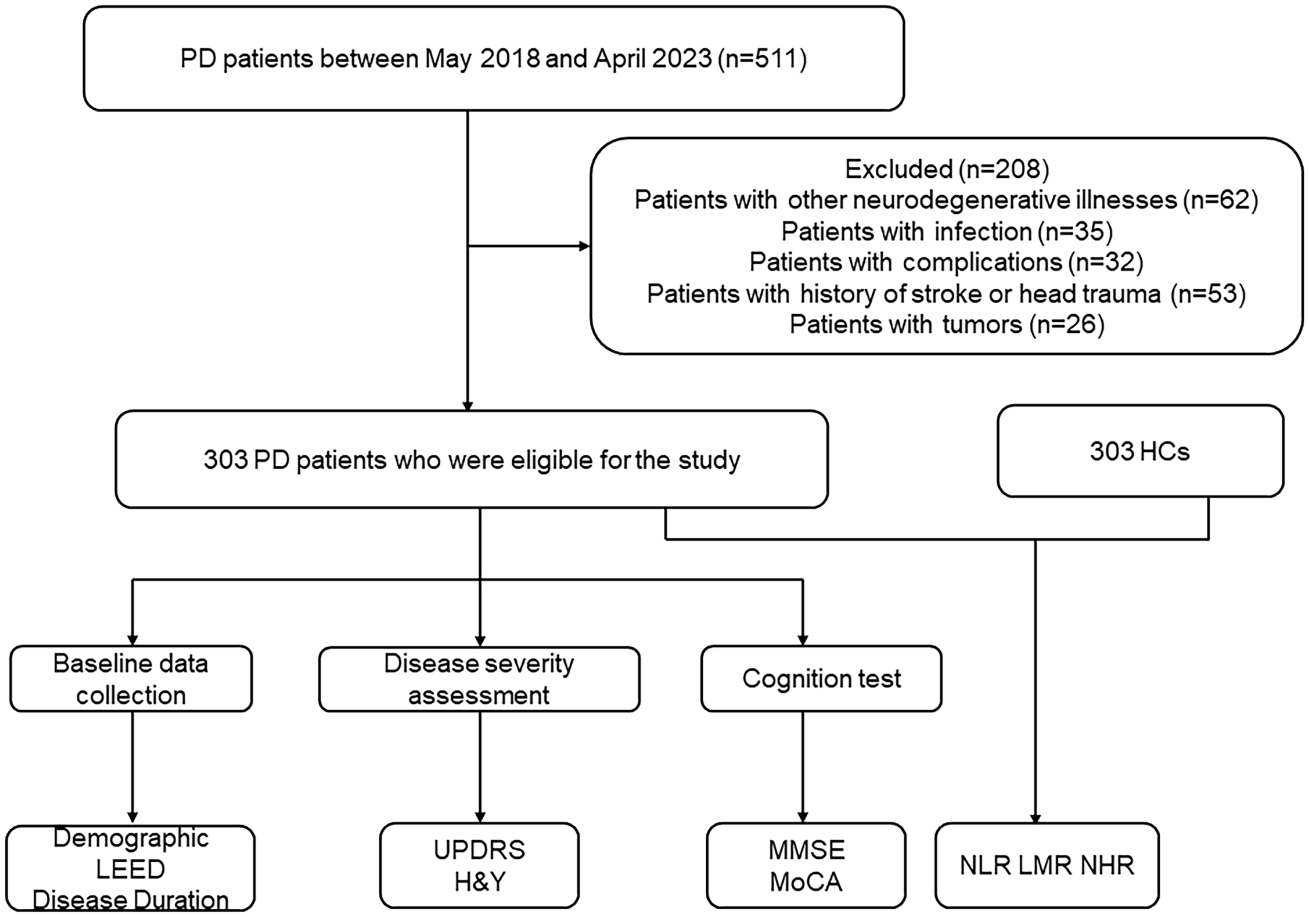
Study flow diagram. PD, Parkinson’s disease; HCs, healthy controls; LEED, levodopa equivalent daily dose; UPDRS, Unified Parkinson’s Disease Rating Scale; H&Y, Hoehn and Yahr staging scale; MoCA, Montreal Cognitive Assessment; MMSE, Mini-Mental State Examination; NLR, neutrophil-to-lymphocyte ratio; LMR, lymphocyte-to-monocyte ratio; NHR, neutrophil-to-high-density-lipoprotein ratio.

Each participant gave permission for the researcher to measure the quality of their blood sample by signing an informed consent form. The inclusion criteria for patients were as follows: (1) had comprehensive relevant clinical and laboratory examination data and (2) met the International Parkinson’s Disease and Movement Disorders Association’s diagnostic criteria (33). The following conditions were considered exclusion criteria: (1) had Alzheimer’s disease (AD), frontotemporal dementia, or any other neurodegenerative illnesses; (2) had blood immune system diseases, tumors, hypothyroidism or acute infection; (3) had complications, such as severe liver and kidney injuries; (4) had a history of cerebral ischemia, transient ischemic attack, intracranial hemorrhage or intracranial injury; and (5) had hypertension, cerebrovascular illness, diabetes mellitus, mental disorders, or statin therapy. Participants serving as healthy controls were recruited from the physical examination facility of the Zhujiang Hospital of Southern Medical University, and the same exclusion criteria were applied. A total of 303 PD patients were recruited between May 2018 and April 2023 (Figure 1).

### Blood collection and clinical evaluation

Clinical assessments were performed in a blinded fashion by experienced neurologists. All patients with PD completed the following standard assessment measures: levodopa equivalent daily dose (LEDD), suitable demographic forms, and the Unified Parkinson’s Disease Rating Scale (UPDRS) score, which includes the UPDRS-I, UPDRS-II, and UPDRS-III subscales, respectively, to assess psychological state, daily routines, and motor function. H&Y was used to measure clinical stages and disease progression of PD patients. In addition, the Montreal Cognitive Assessment (MoCA) and the Mini-Mental State Examination (MMSE) were also applied to determine the patients’ cognitive function associated with PD.

The basic information of each participant, including age, sex, height, and weight, was recorded. Blood samples from all patients were collected at 6–7 a.m. following an 8 h fasting period. Two milliliters of EDTA-anticoagulated whole blood was used for routine blood tests (automated hematology analyzer, XN-10, Japan). Five milliliters of coagulant-containing blood was used for standard biochemical examination (automatic analyzer, HITACHI 7600, Japan). All tests were conducted using commercial kits, which were operated by qualified professionals according to the manufacturer’s specifications. The formula for calculating body mass index (BMI) was weight divided by height squared (kg/m^2^). The counts of white blood cells (WBC), neutrophils, lymphocytes, monocytes, and platelets, as well as the levels of red blood cells (RBC), hemoglobin (Hb), and HDL-C, were assessed in blood samples. The NLR, LMR and NHR were calculated. The testing of blood was repeated three times for each blood test result.

### Statistical analyses

Data analysis was performed by using SPSS 25.0 (IBM SPSS Statistics software, Version 25.0). All the data were tested for a normal distribution using the Kolmogorov–Smirnov test. Continuous variables were presented as mean ± SD, if they were normally distributed; otherwise, they were presented as median (quartile). The results for categorical variables are presented as percentages. The statistical significance of differences between the groups was assessed using the nonparametric Mann–Whitney U test and Kruskal–Wallis test when the data were not normally distributed; student’s *t*-test was used when the data were normally distributed. Spearman rank correlation or Pearson correlation analyses were carried out to investigate the correlation between the various clinical markers and PD. To observe the diagnostic performance of clinical indicators for PD, a receiver operating characteristic (ROC) curve was generated. When *p* < 0.05, statistical significance was inferred.

## Results

### Clinical and demographic characteristics of PD patients and HCs

The demographic and clinical characteristics of the patients are comprehensively described in Table 1. This study included 303 patients with PD [163 males (53.8%) and 140 females (46.2%)] and 303 HCs [142 males (46.8%) and 161 females (53.2%)]. In 59.08% of the PD patients (*n* = 179), onset presented with the AR/PIGD subtype, 12.54% (*n* = 38) presented with the TD subtype as the onset form, and 28.38% (*n* = 86) presented with the mixed subtype at onset. The average age of the patients with PD was 65.04 ± 10.40 years, and that of the HCs was 63.78 ± 8.73 years. The average BMI of patients with PD was 23.00 ± 3.14 kg/m^2^, whereas that of HCs was 23.51 ± 2.68 kg/m^2^. There were no significant differences between patients with PD and HCs in age (*p* = 0.106) or BMI (*p* = 0.053). The average H&Y stage of patients with PD was 2.48 ± 0.87. The mean MoCA score was 17.64 ± 6.08, and the mean MMSE score was 24.18 ± 4.84. The mean UPDRS-I score was 3.58 ± 2.51, the mean UPDRS-II score was 13.16 ± 6.96, and the mean UPDRS-III score was 25.65 ± 14.72. The LEDD was 551.46 ± 261.97.

**Table 1:** Characteristics of PD patients and HCs.

	HCs (*n* = 303)	PD patients (*n* = 303)	*Z*/*T*	*p*
Sex (male, %)	142 (46.8%)	163 (53.8%)	3.492	0.062
Age (years)	63.78 ± 8.73	65.04 ± 10.40	−1.62	0.106
BMI		23.00 ± 3.14	1.939	0.053
Duration (years)		5.21 ± 4.35		
**PD-subtype, No. (%)**				
AR/PIGD subtype		179 (59.08)		
TD subtype		38 (12.54)		
Mixed subtype		86 (28.38)		
UPDRS		42.39 ± 21.91		
UPDRS (I)		3.58 ± 2.51		
UPDRS (II)		13.16 ± 6.96		
UPDRS (III)		25.65 ± 14.72		
H&Y		2.48 ± 0.87		
MMSE		24.18 ± 4.84		
MoCA		17.64 ± 6.08		
LEDD, mg		551.46 ± 261.97		
WBC (×10^9^/L)	5.98 ± 1.27	6.26 ± 1.45	−2.40	0.014
Neutrophils (×10^9^/L)	3.11 ± 0.85	3.74 ± 1.10	−7.657	<0.001
Lymphocytes (×10^9^/L)	2.17 ± 0.54	1.81 ± 0.57	7.891	<0.001
Platelets (×10^12^/L)	233.16 ± 58.80	227.94 ± 56.38	1.116	0.265
Monocytes (×10^9^/L)	0.48 ± 0.14	0.49 ± 0.16	−0.843	0.399
RBC (×10^12^/L)	4.42 ± 0.48	4.39 ± 0.58	0.767	0.443
Hb (g/L)	131.11 ± 12.87	130.69 ± 14.37	0.378	0.705
HDL-C (mmol/L)	1.26 ± 0.34	1.25 ± 0.32	−1.016	0.310
NLR	1.49 ± 0.50	2.26 ± 1.03	−11.776	<0.001
LMR	4.84 ± 1.57	3.95 ± 1.41	7.344	<0.001
NHR	2.71 ± 1.21	3.21 ± 1.93	−3.183	<0.001

BMI, body mass index; WBC, white blood cells; RBC, red blood cells; Hb, hemoglobin; HDL-C, high-density lipoprotein cholesterol; AR/PIGD, akinetic-rigid/postural instability gait difficulty; TD, tremor-dominant; the data shown are the means ± SD; a comparison of variables between PD patients and HCs was performed by student’s *t*-test; *p* < 0.05 was considered to indicate statistical significance.

### Plasma marker comparisons between PD patients and HCs

**Figure 2:**
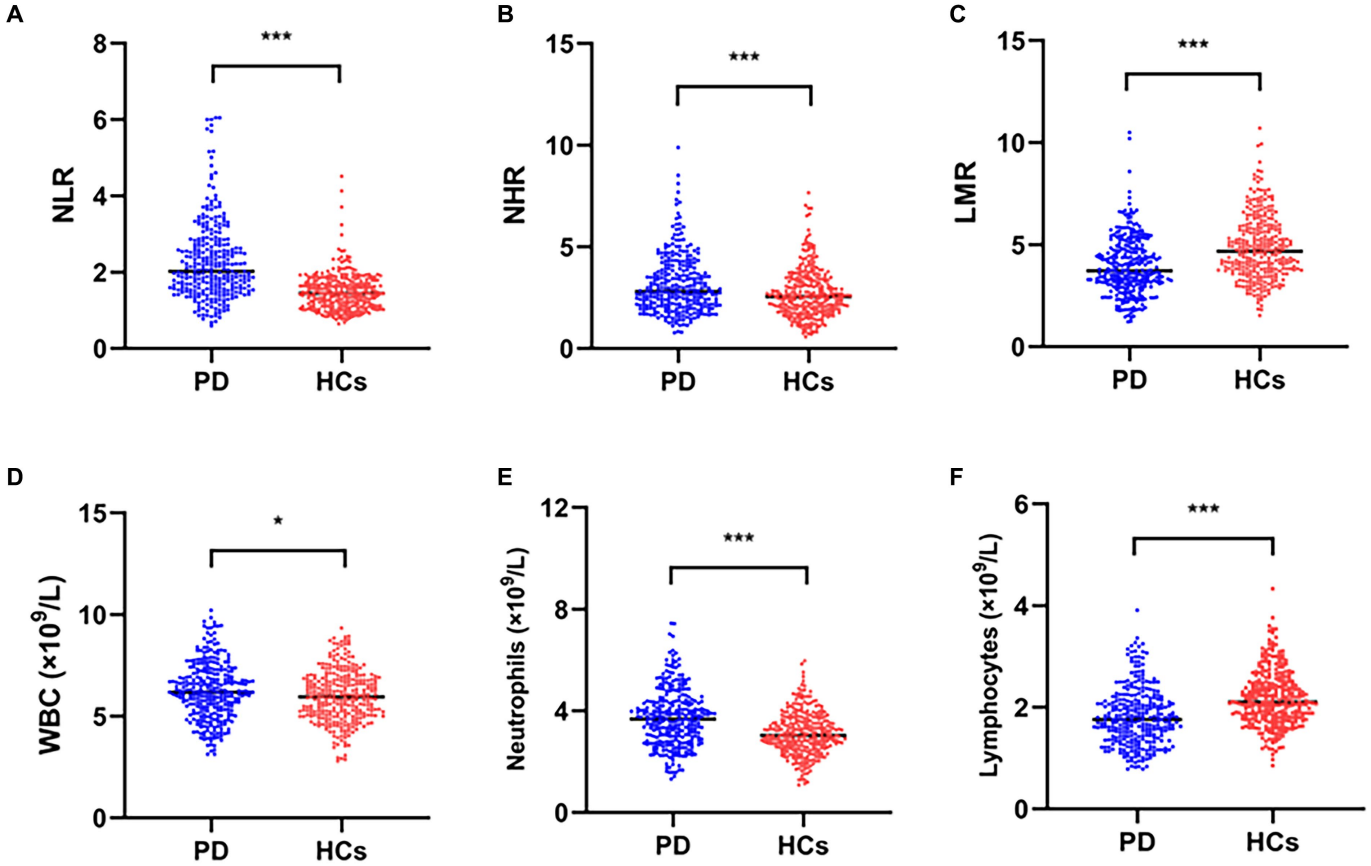
Comparisons of plasma biomarker levels between PD patients and HCs. Comparisons of the NLR **(A)**, NHR **(B)**, LMR **(C)**, WBC **(D)**, neutrophil **(E)** and lymphocyte **(F)** counts between PD patients and HCs; ^*^p < 0.05, ^**^p < 0.01, ^***^p < 0.001.

The results are shown in Table 1 and Figure 2. There were substantial differences between the plasma WBC and neutrophil counts and between the PD patients and HCs in terms of the lymphocyte count, NLR, LMR, and NHR (*p* < 0.05). Patients with PD had substantially lower plasma lymphocyte counts and LMRs than HCs (*p* < 0.05). In addition, the plasma WBC count, neutrophil count, NLR, and NHR were significantly elevated in patients with PD than in HCs (*p* < 0.05).

### PD patients correlation analysis of the UPDRS, H&Y, MoCA, MMSE, NLR, LMR, NHR, neutrophil count, lymphocyte count, and WBC count

**Figure 3:**
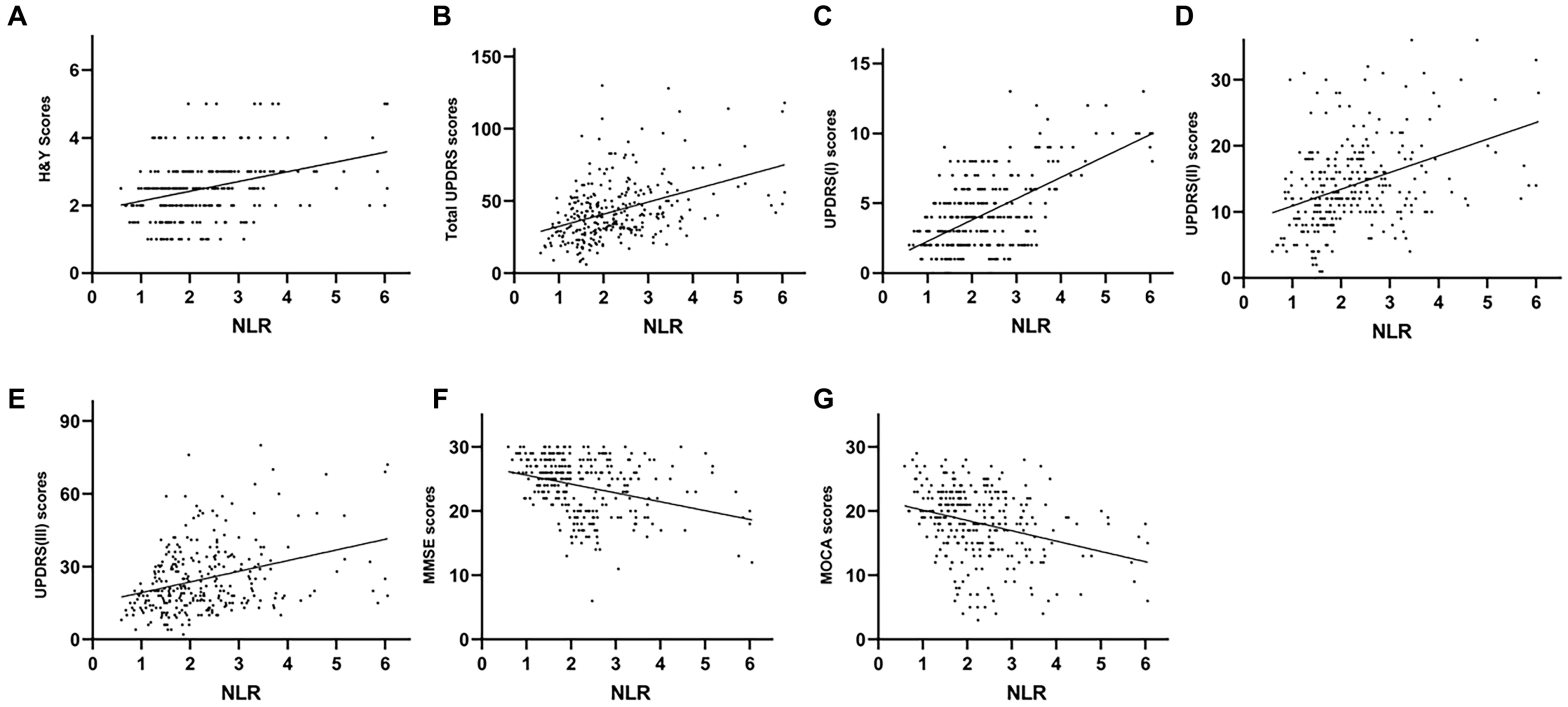
Correlation analysis between the plasma NLR and UPDRS/H&Y/MoCA/MMSE scores in PD patients. Scatter plots showing that the plasma NLR was positively correlated with H&Y (*r* = 0.351, *p* < 0.001; **A**), UPDRS (*r* = 0.426, *p* < 0.001; **B**), UPDRS-I (*r* = 0.445, *p* < 0.001; **C**), UPDRS-II (*r* = 0.345, *p* < 0.001; **D**) and UPDRS-III (*r* = 0.340, *p* < 0.001; **E**) scores and was negatively correlated with MMSE (*r* = −0.316, *p* < 0.001; **F**) and MoCA (*r* = −0.319, and *p* < 0.001; **G**) scores.

**Figure 4:**
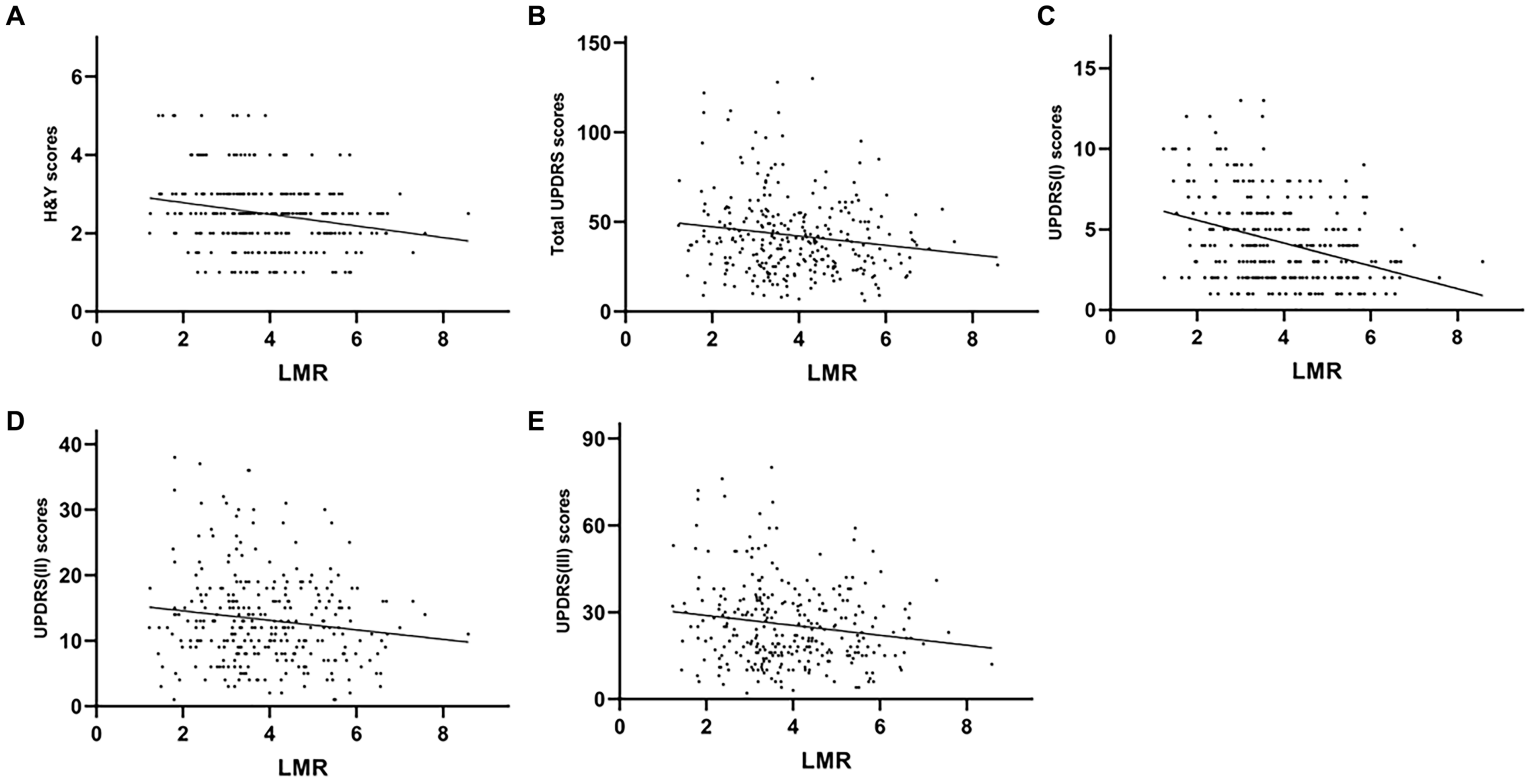
Correlation analysis between the plasma LMR and UPDRS-H&Y scores in PD patients. Scatter plots showing that the plasma LMR was negatively correlated with H&Y (*r* = −0.214, *p* < 0.001; **A**), UPDRS (*r* = −0.347, *p* < 0.001; **B**), UPDRS-I (*r* = −0.318, *p* < 0.001; **C**), UPDRS-II (*r* = −0.255, *p* < 0.001; **D**), and UPDRS-III (*r* = −0.287, *p* < 0.001; **E**) scores.

**Figure 5:**
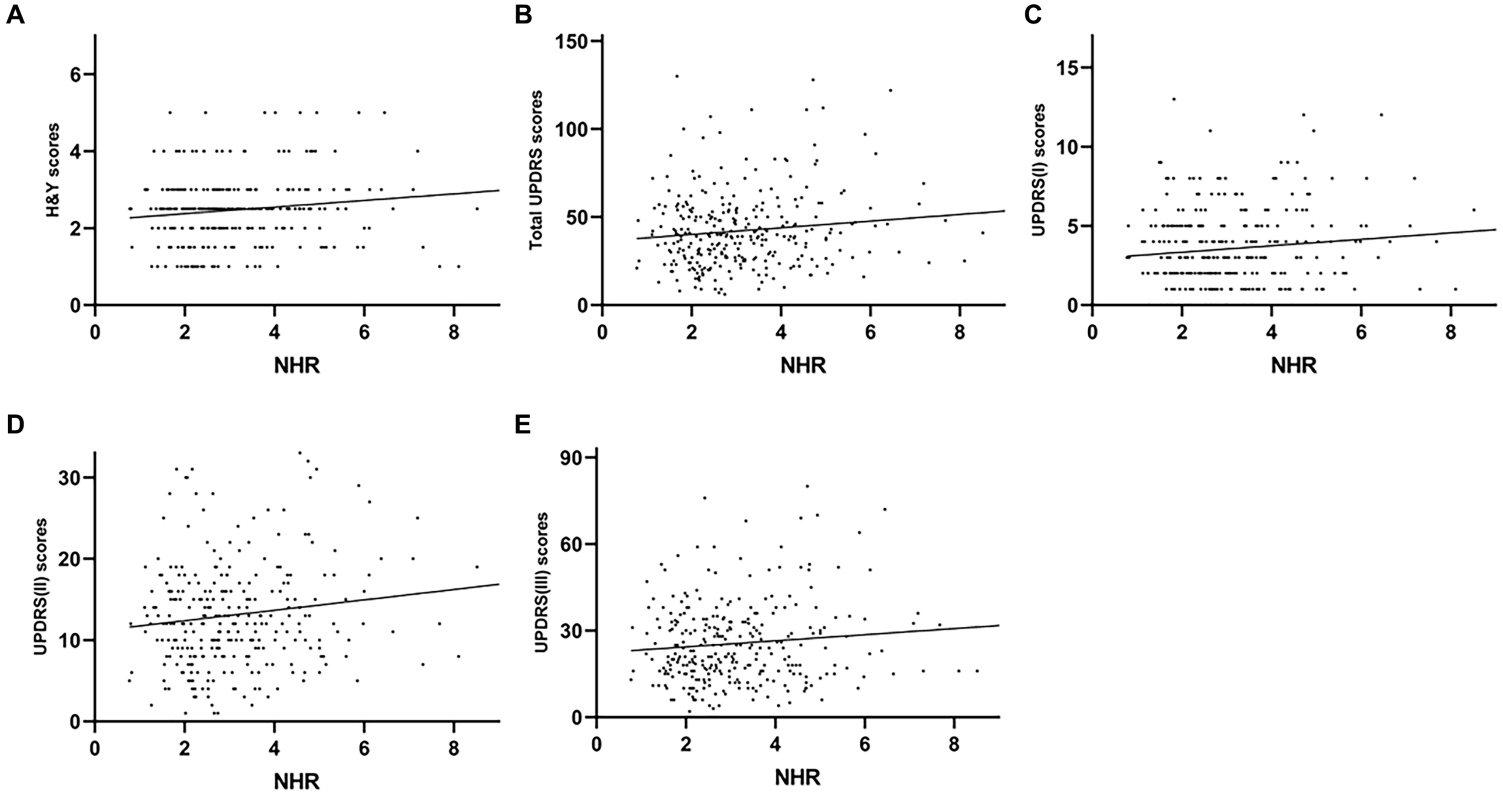
Correlation analysis between the plasma NHR and UPDRS/H&Y scores in PD patients. Scatter plots showing that the plasma NHR was positively correlated with H&Y (*r* = 0.118, *p* = 0.041; **A**), UPDRS (*r* = 0.215, *p* < 0.001; **B**), UPDRS-I (*r* = 0.196, *p* = 0.001; **C**), UPDRS-II (*r* = 0.191, *p* = 0.001; **D**), and UPDRS-III (*r* = 0.170 *p* = 0.003; **E**) scores.

The plasma NLR was positively correlated with the H&Y (*r* = 0.351, *p* < 0.001), UPDRS (*r* = 0.426, *p <* 0.001), UPDRS-I (*r* = 0.445, *p* < 0.001), UPDRS-II (*r* = 0.345, *p* < 0.001) and UPDRS-III (*r* = 0.340, *p* < 0.001) scores. However, plasma NLR had a negative correlation with both MMSE (*r* = −0.316, *p* < 0.001) and MoCA (*r* = −0.319, and *p* < 0.001) (Table 2 and Figure 3). The plasma LMR was also negatively correlated with H&Y (*r* = −0.214, *p* < 0.001), UPDRS (*r* = −0.347, *p* < 0.001), UPDRS-I (*r* = −0.318, *p* < 0.001), UPDRS-II (*r* = −0.255, *p* < 0.001), and UPDRS-III (*r* = −0.287, *p* < 0.001) scores (Table 2 and Figure 4). Interestingly, the plasma NHR was positively correlated with H&Y (*r* = 0.118, *p* = 0.041), UPDRS (*r* = 0.215, *p* < 0.001), UPDRS-I (*r* = 0.196, *p* = 0.001), UPDRS-II (*r* = 0.191, *p* = 0.001), and UPDRS-III (*r* = 0.170 *p* = 0.003) scores (Table 2 and Figure 5). In addition, the neutrophil count was positively correlated with the H&Y score (*r* = 0.163, *p* = 0.022), while the lymphocyte count was negatively correlated with the H&Y score (*r* = −0.182, *p* = 0.001). These findings suggest that an elevated NLR and NHR, as well as a decreased LMR, are associated with the progression and severity of PD.

**Table 2:** Correlation analysis of all variables in PD patients.

Variable	NLR		LMR		NHR		WBC		Neutrophils		Lymphocytes	
	*r*	*p*	*r*	*p*	*r*	*p*	*r*	*p*	*r*	*p*	*r*	*p*
H&Y	0.351	<0.001	−0.214	<0.001	0.118	0.041	0.056	0.032	0.163	0.005	−0.182	0.001
UPDRS	0.426	<0.001	−0.347	<0.001	0.215	<0.001	0.061	0.291	0.104	0.072	−0.092	0.111
(I)	0.445	<0.001	−0.318	<0.001	0.196	0.001	0.080	0.167	0.103	0.075	−0.010	0.868
(II)	0.345	<0.001	−0.255	<0.001	0.191	0.001	0.115	0.046	0.143	0.013	−0.057	0.321
(III)	0.340	<0.001	−0.287	<0.001	0.170	0.003	0.013	0.823	0.056	0.335	−0.111	0.054
MMSE	−0.316	<0.001	0.103	0.073	−0.103	0.075	−0.049	0.392	−0.099	0.085	0.093	0.108
MOCA	−0.319	<0.001	0.074	0.198	−0.094	0.102	−0.009	0.046	−0.062	0.823	0.122	0.034

Pearson correlation was performed to evaluate the relationship between two continuous variables; Spearman’s rank correlation was performed to evaluate the relationship between two continuous or ordinal variables; *p* < 0.05 was considered to indicate statistical significance.

### ROC curves for the NLR, LMR and NHR in the diagnosis of PD

**Figure 6:**
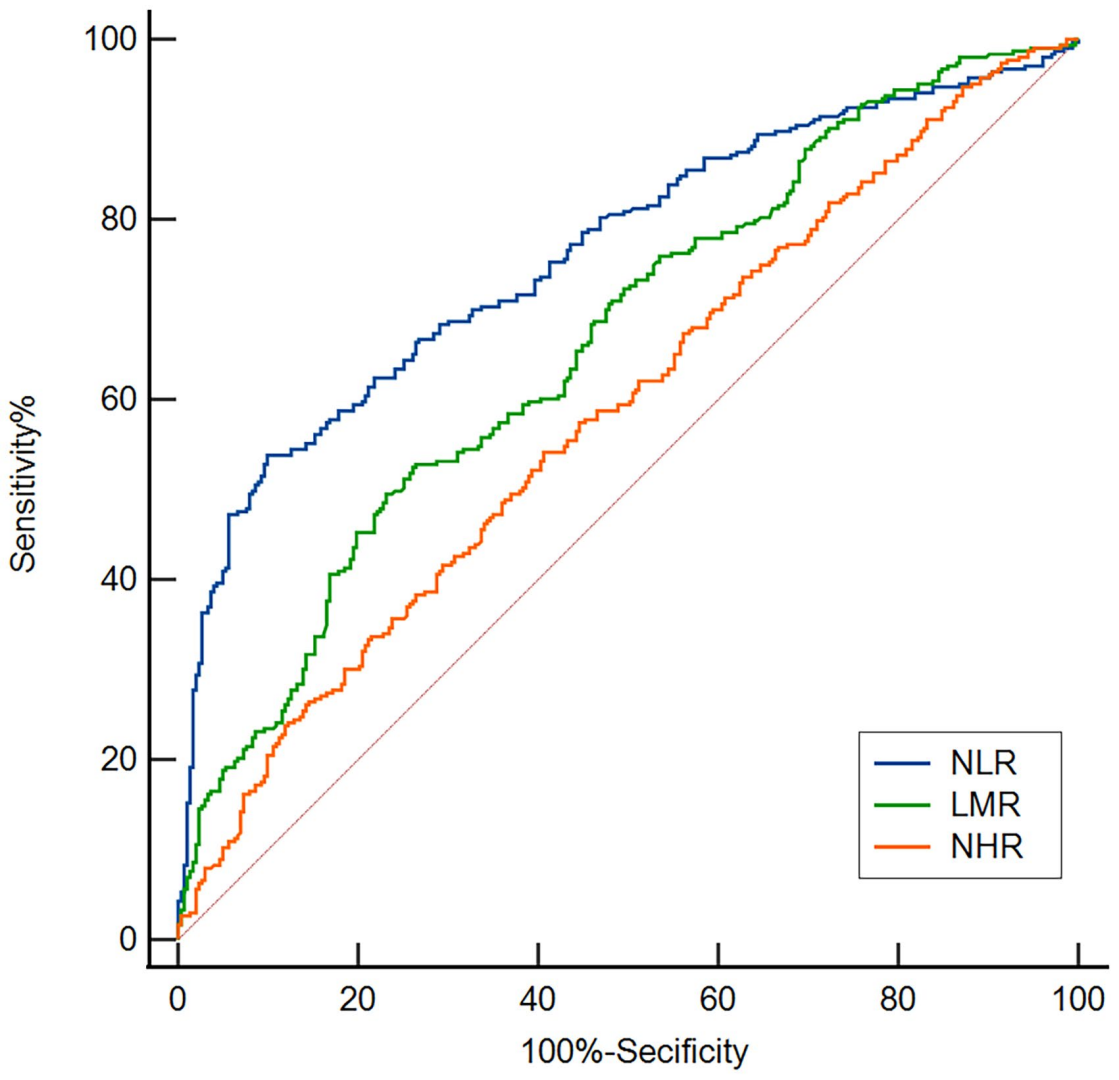
ROC curves for evaluating the utility of the plasma NLR, LMR, and NHR in discriminating PD patients from HCs. Based on the ROC curve analysis, the NLR, LMR and NHR exhibited respectable PD discriminating power, with AUC values of 0.763, 0.665 and 0.588, respectively.

We employed receiver operating characteristic (ROC) curve analysis to examine the utility of the plasma NLR, LMR, and NHR in differentiating patients with PD from HCs. For the NLR, the AUC was 0.763 (95% CI: 0.727–0.796, *p* < 0.001), the cutoff was 1.94, and the sensitivity and specificity were 53.8% and 90.1%, respectively (Figure 6). For the LMR, the AUC was 0.665 (95% CI: 0.626–0.702, *p* < 0.001), the cutoff was 3.67, and the sensitivity and specificity were 49.5 and 76.9%, respectively (Figure 6). For the NHR, the AUC was 0.588 (95% CI: 0.548–0.628, *p* < 0.001), the cutoff was 2.70, and the sensitivity and specificity were 54.13 and 59.41%, respectively (Figure 6).

## Discussion

Our analysis focused on the variation in plasma NLR, LMR, and NHR among individuals with PD. Initially, we observed significantly elevated plasma NLR and NHR in patients with PD compared with HCs, while plasma NHR were considerably lower. Furthermore, we discovered that elevated plasma NLR and NHR, as well as decreased plasma LMR, were closely related to the severity of PD. Finally, our study elucidated the utility of the NLR, LMR, and NHR as potential markers for differentiating PD patients from HCs.

A growing body of evidence supports the role of peripheral inflammatory conditions in the pathophysiology of PD (34). This involves not only cytokines but also peripheral immune cells (7, 8, 35–37). Concerning peripheral inflammatory markers, cytokines, which communicate with modulate peripheral and central immune compartments, appear to be dysregulated in the blood, cerebrospinal fluid, and brain of patients with PD (35, 37, 38). Additionally, leukocytes and their subpopulations are also reported to be quantitatively and qualitatively altered in peripheral blood (23, 36, 39). Furthermore, inflammatory factors or immune cells abnormally activated in the periphery may infiltrate the central nervous system, thereby potentially triggering inflammation within the central nervous system, which could result in the degeneration of dopaminergic neurons (40, 41). In summary, peripheral inflammation is crucial in the pathogenesis of PD. Therefore, it was necessary for us to evaluate the association between peripheral inflammation and the severity of PD.

From several studies, scholars have reported that increased permeability of the BBB occurs in patients with advanced-stage PD (42), and these findings contribute to the pathophysiology of PD (43). As a result of BBB impairment, the neutrophil and monocyte counts increase when peripheral inflammation occurs and are correlated with the worsening of clinical symptoms in patients with PD (28, 44). Interestingly, previous research has demonstrated that nonsteroidal anti-inflammatory drugs (NSAIDs) can reduce the neutrophil count; therefore, NSAIDs may be useful for the prevention and treatment of PD (45). Our previous study also showed that Dl-3-n-butylphthalide rescues dopaminergic neurons in PD models by inhibiting the NLRP3 inflammasome and ameliorating mitochondrial impairment (46). Moreover, our study revealed that patients had considerably higher neutrophil counts than HCs, which confirms, to some extent, the involvement of neutrophils in PD pathogenesis. Consistent with the findings of prior research, in our study, we discovered that the plasma lymphocyte count in patients with PD was considerably lower than that in HCs (23, 47). One study revealed that a reduced lymphocyte level in plasma is related to an increased risk of PD (21). CD8^+^ and CD4^+^ T lymphocytes enter the brain in the 1-methyl-4-phenyl-1,2,3,6 tetrahydropyridine (MPTP) PD animal model and postmortem PD specimens (48), and lower plasma lymphocyte counts in patients with PD may be linked to BBB malfunction, which results in lymphocyte recruitment to the brain parenchyma. In addition, the NLR could serve as a peripheral indicator of the level of systemic inflammation (23), as this ratio incorporates information from both leukocyte subsets and supplementary immunological pathways.

Our study revealed a significantly higher NLR in the plasma of PD patients than in HCs. The results also highlighted a correlation between plasma NLR and the total UPDRS scores as well as the UPDRS I-III scores in patients with PD. These findings indicate a possible association between plasma NLR and the progression of PD, which is consistent with the findings of a previous study (24). However, other studies have not shown a relationship between the NLR and the severity of PD (23). In addition, our study showed that there were no significant differences in the NLR among patients with AR/PIGD, TD or mixed subtypes, which is consistent with a previous study (49). The differences among various studies may stem from variations in the study population’s ethnicity, limited sample size, medication status, disease duration and disease severity. Additionally, we noted a substantial negative relationship between the plasma NLR and MoCA and MMSE scores, suggesting that the NLR may be connected to the pathophysiological mechanism of cognitive impairment. Interleukin-6 (IL-6), tumor necrosis factor-a (TNF-a), and C-reactive protein have been shown to be linked to cognitive impairment (50), and changes in peripheral CD4^+^, CD8^+^, CD3^+^, and CD4^+^/CD3^+^ levels have been documented (51, 52). Animal experiments have shown that neutrophils can promote AD pathology and cognitive decline, and depletion of neutrophils has the important ability to ameliorate the neuropathological features of disease and memory deficits (53). Additionally, markers related to peripheral blood neutrophils can predict a decrease in executive function in patients with AD (54). A recent study indicated that an elevated NLR is associated with cognitive impairment in PD patients (55). Therefore, we explored the potential of the NLR as a biomarker. Our analysis used the ROC curve and yielded an AUC of 0.763, with a sensitivity of 53.8% and a specificity of 90.1%. These results affirm the value of NLR as a prognostic indicator, which is consistent with prior research (24, 56). According to our findings, the NLR could be a crucial factor for determining the severity of PD.

The NHR is the ratio of neutrophils to HDL-C. It has the benefit of representing the complementary link between multiple routes and can more accurately reflect peripheral immune inflammatory alterations than single indicators. A study reported that an increased NHR was observed in patients with acute ischemic stroke who underwent thrombolytic therapy when the degree of nerve injury increased (57). Moreover, a prior study revealed a negative correlation between the NHR and PD incidence, suggesting that the NHR was an accurate indicator of the inflammatory process in PD patients (29). Interestingly, our findings indicated a significant increase in the plasma NHR in patients with PD compared with HCs. Moreover, there were positive correlations between plasma NHR and H&Y scores, UPDRS total scores and UPDRS I-III scores. However, our investigation demonstrated a low AUC for the NHR, which means it is poorly predictive in the diagnosis of PD. Therefore, additional future research is required. Nevertheless, the NHR remains a promising measure for evaluating the severity of PD.

A recent study has shown that α-synuclein has a proinflammatory effect on monocytes, and elevated monocyte levels are considered an indicator of the severity of PD (30). Interestingly, previous research suggests that early decrease of peripheral blood intermediate monocytes is characteristic of multiple system atrophy-cerebellar type (MSA-C) and is a biomarker (58). In addition, it has been previously shown that monocytes and their precursors are upregulated in the peripheral blood of PD patients (59). This study revealed that the plasma mononuclear cell count did not significantly differ between PD patients and HCs. This could be attributed to the fact that the patients with PD who participated in this study were receiving dopamine-based drug treatments (60). The LMR is considered a good inflammatory marker and has been widely used in cerebrovascular diseases and primary and secondary tumors (61). We investigated the correlation between the plasma LMR and the severity of PD. Our findings revealed a significant reduction in the plasma LMR among patients with PD in comparison to HCs. Furthermore, these disparities exhibited negative correlations with respect to H&Y, UPDRS, UPDRS-II, and UPDRS-III scores. Our findings demonstrated an AUC of 0.665 along with a sensitivity of 49.5% and specificity of 76.9%. Thus, the LMR may be utilized as a marker for assessing the severity of PD.

Inflammation is important in the pathogenesis of PD. The NLR, LMR and NHR are considered highly sensitive markers of inflammation. This study revealed that the NLR is a reliable predictor of the severity of PD, and the NHR also has the potential to serve as an assessment indicator. In addition, for the first time, we found that low LMR was associated with the severity of PD. To conclude, we found that the NLR and LMR have better discriminatory ability for PD. The use of these markers in clinical practice has the potential to increase the accuracy of PD diagnosis and treatment. Furthermore, these markers are convenient and cost effective and require minimal sampling effort, making them readily applicable for large-scale replication.

This study has the following limitations: (1) it was a cross-sectional study, and our findings indicated that the NLR, LMR, and NHR were weakly associated with the severity of PD, which may be attributable to the larger sample size; hence, longitudinal cohort studies with larger populations are needed in the future; (2) there is a lack of other inflammatory markers available to further assess whether the NLR, LMR, and NHR correlate with other inflammatory markers, preventing us from having a comprehensive understanding of the role inflammation plays in PD; and (3) the NLR, LMR, and NHR are recognized as markers of inflammation in the peripheral circulation; they are not specific neuroinflammatory markers and can be influenced by various factors, thus making it challenging to draw conclusions about their specific impact on certain neuroinflammatory pathways.

In conclusion, our findings suggest a correlation between NLR, LMR, NHR and the severity of PD. In addition, the NLR, LMR, and NHR may be effective biomarkers of PD and could be used to predict the severity of PD; moreover, these biomarkers have great clinical importance for the diagnosis and severity of PD. However, future studies are necessary to validate these results and to further understand the pathogenesis of PD.

## Data availability statement

The raw data supporting the conclusions of this article will be made available by the authors, without undue reservation.

## Ethics statement

The studies involving humans were approved by the Zhujiang Hospital of Southern Medical University’s Ethics Committee granted approval for this study (2022-KY-186). The studies were conducted in accordance with the local legislation and institutional requirements. The participants provided their written informed consent to participate in this study.

## Author contributions

FYL: Data curation, Formal analysis, Investigation, Methodology, Software, Writing – original draft. GMW: Data curation, Formal analysis, Investigation, Methodology, Writing – original draft. HZ: Data curation, Formal analysis, Investigation, Methodology, Project administration, Software, Supervision, Writing – original draft, Writing – review & editing. WJZ: Data curation, Formal analysis, Investigation, Methodology, Writing – review & editing. BD: Data curation, Formal analysis, Investigation, Methodology, Supervision, Writing – review & editing. YQL: Data curation, Investigation, Methodology, Writing – review & editing. XT: Writing – review & editing, Conceptualization, Methodology, Supervision. MZD: Formal analysis, Investigation, Writing – review & editing, Data curation. HQG: Investigation, Methodology, Writing – review & editing, Formal analysis. SSZ: Investigation, Methodology, Supervision, Writing – review & editing, Resources. QW: Data curation, Funding acquisition, Investigation, Methodology, Project administration, Supervision, Validation, Writing – review & editing.
